# Small Heat Shock Proteins, Big Impact on Protein Aggregation in Neurodegenerative Disease

**DOI:** 10.3389/fphar.2019.01047

**Published:** 2019-09-18

**Authors:** Jack M. Webster, April L. Darling, Vladimir N. Uversky, Laura J. Blair

**Affiliations:** Department of Molecular Medicine, USF Byrd Institute, University of South Florida, Tampa, FL, United States

**Keywords:** neurodegeneration, proteostasis, molecular chaperone, sHsps, HspB, aging

## Abstract

Misfolding, aggregation, and aberrant accumulation of proteins are central components in the progression of neurodegenerative disease. Cellular molecular chaperone systems modulate proteostasis, and, therefore, are primed to influence aberrant protein-induced neurotoxicity and disease progression. Molecular chaperones have a wide range of functions from facilitating proper nascent folding and refolding to degradation or sequestration of misfolded substrates. In disease states, molecular chaperones can display protective or aberrant effects, including the promotion and stabilization of toxic protein aggregates. This seems to be dependent on the aggregating protein and discrete chaperone interaction. Small heat shock proteins (sHsps) are a class of molecular chaperones that typically associate early with misfolded proteins. These interactions hold proteins in a reversible state that helps facilitate refolding or degradation by other chaperones and co-factors. These sHsp interactions require dynamic oligomerization state changes in response to diverse cellular triggers and, unlike later steps in the chaperone cascade of events, are ATP-independent. Here, we review evidence for modulation of neurodegenerative disease-relevant protein aggregation by sHsps. This includes data supporting direct physical interactions and potential roles of sHsps in the stewardship of pathological protein aggregates in brain. A greater understanding of the mechanisms of sHsp chaperone activity may help in the development of novel therapeutic strategies to modulate the aggregation of pathological, amyloidogenic proteins. sHsps-targeting strategies including modulators of expression or post-translational modification of endogenous sHsps, small molecules targeted to sHsp domains, and delivery of engineered molecular chaperones, are also discussed.

## Introduction

Maintenance of cellular protein homeostasis (proteostasis) is crucial for cell function and survival (Powers et al., 2009; Klaips et al., 2018; Yu et al., 2019). Neurons are particularly sensitive to dysregulated proteostasis as evidenced by the accumulation and aggregation of amyloidogenic proteins, which are a hallmark of neurodegenerative disease (Skibinski and Finkbeiner, 2013; Smith et al., 2015; Yerbury et al., 2016). Proteostasis is regulated by networks of interacting proteins that include translation machinery (Steffen and Dillin, 2016; Anisimova et al., 2018), secretory pathways (Plate and Wiseman, 2017; Remondelli and Renna, 2017; Wong et al., 2018), proteases (Wilson et al., 2015; Klein et al., 2018), the ubiquitin-proteasome system (UPS) (Bett, 2016), autophagic machinery (Carra et al., 2009; Menzies et al., 2015), and molecular chaperones (Balchin et al., 2016). In addition to their vital role in folding nascent proteins, molecular chaperones recognize and triage proteins if misfolding events occur. Chaperones can be classified as holdases (that bind and hold partially folded protein intermediates to prevent their aggregation), foldases (that assist the proper folding of proteins in an ATP-dependent manner), and unfoldases (that convert misfolded proteins into transiently unfolded intermediates to provide an opportunity for spontaneous proper refolding) (Hoffmann et al., 2004; Mattoo and Goloubinoff, 2014). ATP-dependent chaperones, like the 70 kDa heat shock protein (Hsp70) and the 90 kDa heat shock protein (Hsp90), facilitate refolding, degradation, or sequestration of these misfolded proteins. In mammalian systems, chaperones often work together in multisubunit heterocomplexes, comprised of an ATP-dependent chaperone as well as co-chaperone and accessory proteins. These discrete heterocomplexes allow for specific client selection, the dynamic modulation of these clients by regulating ATPase activity, or, if a protein is unable to refold, delivery to additional proteostasis machinery for degradation or sequestration (Sahasrabudhe et al., 2017; Chen et al., 2018; Freilich et al., 2018; Grousl et al., 2018). Here, we review evidence for modulation of neurodegenerative disease-relevant protein aggregation by a family of molecular chaperones, known as small heat shock proteins (sHsps).

## Structural Plasticity of sHsps

sHsps are a special class of molecular chaperones that lack an ATPase domain. They are characterized as small proteins, between 12 and 43 kDa, containing a core α-crystallin domain (ACD) flanked by variable N-terminal and C-terminal domains (de Jong et al., 1998; Franck et al., 2004; Haslbeck et al., 2005; Mchaourab et al., 2009; Kriehuber et al., 2010; Haslbeck and Vierling, 2015; Treweek et al., 2015; Zhu and Reiser, 2018). There are 10 α-crystallin domain-containing mammalian sHsps, designated HspB1 (Hsp27), HspB2 (myotonic dystrophy kinase-binding protein, MKBP), HspB3 (Hsp17), HspB4 (αA-crystallin), HspB5 (αB-crystallin), HspB6 (Hsp20), HspB7 (cardiovascular Hsp, cvHsp), HspB8 (Hsp22), HspB9 (cancer/testis antigen 51, CT51) and HspB10 (outer dense fiber protein 1, ODFP1) (see **Table 1**) (Kappé et al., 2003; Kampinga et al., 2009). In general, sHsps interact with intermediately folded proteins through surface exposed hydrophobic residues in order to stabilize the protein and prevent further misfolding and/or aggregation (Sharma et al., 1997; van Montfort et al., 2001; Haslbeck et al., 2005; Jaya et al., 2009). Therefore, sHsps act early in the chaperone processing of misfolded proteins, often prior to refolding attempts by ATP-dependent chaperone complexes (Mchaourab et al., 2009; Bakthisaran et al., 2015; Fu, 2015; Haslbeck and Vierling, 2015). The dynamic nature of sHsp structure appears to be an important determinant for client protein binding and chaperone function (Leroux et al., 1997; Jaya et al., 2009; Mchaourab et al., 2009; Peschek et al., 2009; Benesch et al., 2010; Stengel et al., 2010). sHsp dynamics occur at five levels, including through 1) flexible domains flanking the ACD, 2) polydisperse self-multimerization, 3) multimerization with other sHSPs, 4) subunit exchange, and 5) regulation by the cellular environment including post-translational modifications (Fu, 2015), which are discussed in more detail below.

**Table 1:** sHsp expression and relevance for neurodegenerative diseases.

HspB (alias)	Normal brain expression	Stress inducible neuronal expression	Interactions with amyloidogenic proteins	Effect on aggregation	Association with neurodegenerative disease
HspB1 (Hsp27)	Protein (Quraishe et al., 2008) mRNA (Kirbach and Golenhofen, 2011)	Heat (Kirbach and Golenhofen, 2011) Oxidative (Bartelt-Kirbach and Golenhofen, 2014; Bartelt-Kirbach et al., 2017)	Aβ (Ojha et al., 2011b) tau (Shimura et al., 2004; Freilich et al., 2018) α-Syn (Bruinsma et al., 2011; Cox et al., 2018) SOD-1 (Yerbury et al., 2013) TDP-43 (Wan et al., 2015)	↑Aβ (Ojha et al., 2011b) ↓Aβ (Wilhelmus et al., 2006a; Yoshiike et al., 2008) ↓Tau (Abisambra et al., 2010; Baughman et al., 2018; Freilich et al., 2018; Mok et al., 2018) ↓α-Syn (Bruinsma et al., 2011; Cox et al., 2018) ↓ polyQ (Vos et al., 2010)	AD (Wilhelmus et al., 2006c; Björkdahl et al., 2008; Yu et al., 2018) HD (Brehme et al., 2014) PD ( Zhang et al., 2005; Brehme et al., 2014)
HspB2 (MKBP)	mRNA (Kirbach and Golenhofen, 2011)		α-Syn (Bruinsma et al., 2011)	↓α-Syn (Bruinsma et al., 2011)	AD (Wilhelmus et al., 2006c; Yu et al., 2018) HD (Brehme et al., 2014) PD (Brehme et al., 2014)
HspB3 (Hsp17)	mRNA (Kirbach and Golenhofen, 2011)				AD (Brehme et al., 2014)
HspB4 (αA-crystallin)				↓ polyQ (Vos et al., 2010)	
HspB5 (αB-crystallin)	Protein (Quraishe et al., 2008) mRNA (Kirbach and Golenhofen, 2011)	Heat (Kirbach and Golenhofen, 2011) Oxidative (Bartelt-Kirbach and Golenhofen, 2014; Bartelt-Kirbach et al., 2017 )	Aβ (Shammas et al., 2011) α-Syn (Rekas et al., 2004; Waudby et al., 2010; Bruinsma et al., 2011; Cox et al., 2018) polyQ (Robertson et al., 2010) SOD-1 (Yerbury et al., 2013)	↓Aβ (Wilhelmus et al., 2006a; Shammas et al., 2011) ↓α-Syn (Rekas et al., 2004; Waudby et al., 2010; Bruinsma et al., 2011)	AD (Wilhelmus et al., 2006c; Björkdahl et al., 2008) HD (Brehme et al., 2014) PD (Brehme et al., 2014) ALS (Gorter et al., 2018)
HspB6 (Hsp20)	Protein (Quraishe et al., 2008) mRNA (Kirbach and Golenhofen, 2011)	Oxidative (Bartelt-Kirbach and Golenhofen, 2014)	Aβ (Cameron et al., 2014) α-Syn (Bruinsma et al., 2011)	↓Aβ (Wilhelmus et al., 2006a) ↓α-Syn (Bruinsma et al., 2011) ↓ polyQ (Vos et al., 2010)	AD (Wilhelmus et al., 2006c)
HspB7 (cvHsp)	Protein (Uhlén et al., 2015) mRNA (Quraishe et al., 2008)			↓↓ polyQ (Vos et al., 2010)	
HspB8 (Hsp22)	Protein (Quraishe et al., 2008) mRNA (Kirbach and Golenhofen, 2011)	Heat (Kirbach and Golenhofen, 2011) Oxidative (Bartelt-Kirbach and Golenhofen, 2014)	Aβ (Wilhelmus et al., 2006b) α-Syn (Bruinsma et al., 2011)	↓Aβ (Wilhelmus et al., 2006b) ↓ α-Syn (Bruinsma et al., 2011) ↓ polyQ (Vos et al., 2010)	AD (Brehme et al., 2014; Wilhelmus et al., 2006b) HD (Brehme et al., 2014) ALS (Gorter et al., 2018)
HspB9 (CT51)				↓ polyQ (Vos et al., 2010)	
HspB10 (ODFP1)					

*↑*, increased; *↓*, reduced

**Figure 1:**
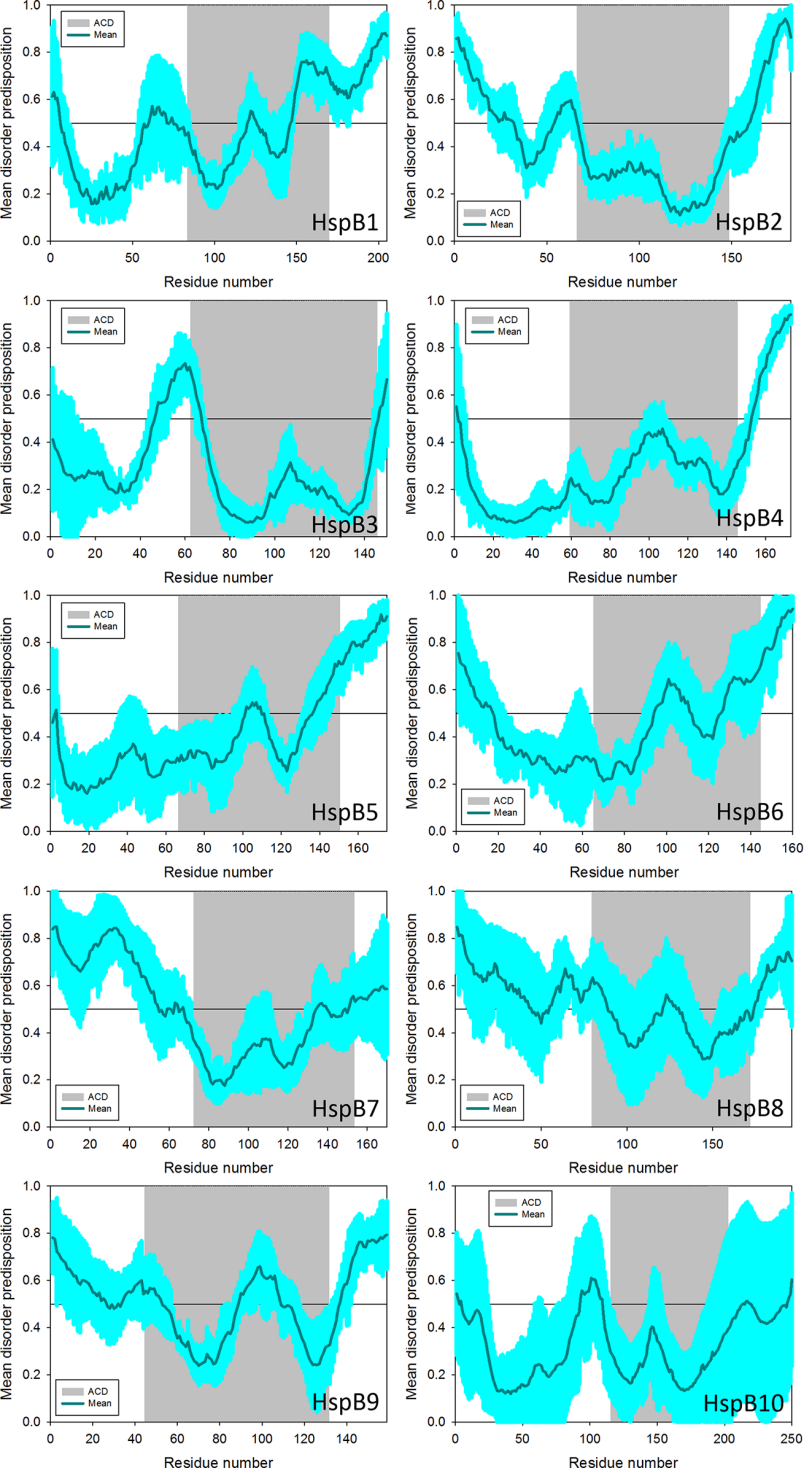
Intrinsic disorder profiles generated for human HspB1 (UniProt ID: P04792), HspB2 (UniProt ID: Q16082), HspB3 (UniProt ID: Q12988), HspB4 (UniProt ID: P02489), HspB5 (UniProt ID: P02511), HspB6 (UniProt ID: O14558), HspB7 (UniProt ID: Q9UBY9), HspB8 (UniProt ID: Q9UJY1), HspB9 (UniProt ID: Q9BQS6), and HspB10 (UniProt ID: Q14990), visualized as the mean of 6 commonly used disorder predictors from the PONDR family, PONDR^®^ VLXT, PONDR^®^ VSL2, PONDR^®^ VL3, PONDR^®^ FIT, IUpred_short and IUpred_long. In these plots, disorder score exceeding the 0.5 threshold correspond to intrinsically disordered regions, whereas disorder score ranging from 0.2 to 0.5 show flexible regions. The light cyan shade around disorder predisposition curves represents error (SD) distribution. The grey shaded background corresponds to the ACD of each sHsp sequence to facilitate discrimination from the terminal domains.

The disordered nature of the N- and C-termini flanking the ACD allows for tertiary flexibility that likely supports dynamic interactions with client proteins as well as other sHsp subunits (Lindner et al., 2000; Stromer et al., 2004; Baldwin et al., 2011; Jehle et al., 2011; Sudnitsyna et al., 2012; Patel et al., 2014; Carver et al., 2017). Observations of intrinsic disorder propensity based on primary structure are presented in **Figure 1**. A universal intrinsic disorder propensity within the C-terminal domains appears to be a common trait among human sHsp family members; while disorder propensity among N-terminal domains are more variable. HspB4 and HspB5 (the α-crystallins) distinguish themselves as having the most ordered N-terminal domains. The N-terminal domains of HspB1, HspB2, HspB3, HspB6, and HspB10 have discrete segments of disorder; while HspB7, HspB8, and HspB9 have a more widespread disorder propensity in the variable N-terminal domain (**Figure 1**). It is notable that, for many of the sHsps, high disorder propensity also extends into the conserved ACDs, which are generally regarded to have a well-structured immunoglobulin-like fold of β-sheets (Bagnéris et al., 2009; Laganowsky et al., 2010; Carver et al., 2017), and may reflect differences in ACD flexibility among sHsps.

sHsps utilize flexible hydrophobic surfaces to interact with exposed hydrophobic surfaces of client proteins, which are made available during misfolding or denaturation (Sharma et al., 1997; van Montfort et al., 2001; Haslbeck et al., 2005; Jaya et al., 2009). Both high affinity and dynamic low affinity interactions of sHsps with client proteins have been described (Bruinsma et al., 2011; McDonald et al., 2012; Mattoo and Goloubinoff, 2014; Ecroyd, 2015). Generally, high affinity interactions are associated with amorphous misfolding clients while sHsp effects on amyloid fibril formation are often due to weak or transient client interactions (Kulig and Ecroyd, 2012; Treweek et al., 2015). Expression of sHsps, with their own exposed hydrophobic surfaces, could potentially pose a cellular proteostasis problem of its own (Dobson, 1999; Seong and Matzinger, 2004; Díaz-Villanueva et al., 2015). One way for cells to address this problem is to restrict expression of sHsps until stress events (like heat shock or oxidative stress) necessitate chaperone activity. Another mechanism is to sequester exposed hydrophobic regions into large, dormant, multimeric structures (sHsp oligomers); keeping sHsp chaperones poised to handle early misfolding events prior to transcription and translation of other stress-inducible chaperones (Santhanagopalan et al., 2018). Indeed large, inactive, oligomeric sHsps disperse into smaller oligomers as they become active (Basha et al., 2012; Haslbeck et al., 2019). This transition can be precipitated by stress events, post-translational modifications, and by direct competition of client proteins with sHsp oligomeric interactions (Arrigo, 2017; Freilich et al., 2018).

Dynamic sHsp homodimers and polydisperse, homooligomeric structures regulate chaperone function as well as client protein specificity and binding (Leroux et al., 1997; Kim et al., 1998; van Montfort et al., 2001; Stromer et al., 2004; Horwitz, 2009; Jaya et al., 2009; Mchaourab et al., 2009; Peschek et al., 2009; Benesch et al., 2010; Jehle et al., 2010; Stengel et al., 2010; Baldwin et al., 2011; Patel et al., 2014). Many factors regulate the size of these structures. sHsp expression (or concentration) itself alters the size of oligomers *in vitro* (Shashidharamurthy et al., 2005). HspB1 and HspB5 each form higher order homooligomers of greater than 20 subunits *in vitro*, while other family members generally form smaller oligomers or dimers (Mymrikov et al., 2017). Moreover, sHsp homooligomers with a discrete number of subunits may actually be composed of a variety of dynamic structures, since polymorphic arrangement of the dimer and oligomer subunits have been reported (Feil et al., 2001; Hochberg et al., 2014; Fu, 2015).

Chaperone activity is also regulated by the formation of dynamic sHsp heterodimers and heterooligomers that undergo continuous subunit exchange (Bova et al., 2000; Mchaourab et al., 2009; Aquilina et al., 2013; Arrigo, 2013). Native α-crystallin in the eye lens is made up of polydisperse HspB4/HspB5 heterooligomeric complexes (Haley et al., 2000; Shi et al., 2006; Horwitz, 2009). The crystal structure of a tetrameric HspB2/HspB3 heterocomplex, which is found in muscle cells, was recently reported (den Engelsman et al., 2009; Clark et al., 2018). While a complete picture of the physiologically relevant heterooligomeric sHsp combinations has not been elucidated, *in vitro* evidence for a variety of heterooligomeric sHsp groupings is emerging, including: HspB1/HspB5 (Zantema et al., 1992; Aquilina et al., 2013), HspB1/HspB6 (Boros et al., 2004; Bukach et al., 2009; Heirbaut et al., 2017) and HspB2/HspB6 (Boros et al., 2004) heterooligomeric complexes as well as HspB8 interactions with HspB1, HspB2, HspB5, HspB6, and HspB7 (Sun et al., 2004; Fontaine et al., 2005). The relative expression levels of discrete sHsp family members will influence the complement of oligomers resulting from subunit exchange (Bova et al., 2000; Mchaourab et al., 2009; Aquilina et al., 2013; Arrigo, 2013), which may guide an interminable number of oligomeric combinations.

Changes in the cellular environment and post-translational modifications also regulate chaperone activity by affecting the structural and multimerization dynamics of sHsps (Eaton et al., 2002; Nagaraj et al., 2003; Mymrikov et al., 2011). Environmental influencers include alterations in temperature and pH (Lelj-Garolla and Mauk, 2006; Hayes et al., 2009; Clouser and Klevit, 2017). The most commonly studied post-translational modification of sHsps is phosphorylation within the N-terminal domain, which is generally considered to decrease oligomer size (Ito et al., 1997; van den IJssel et al., 1998; Lambert et al., 1999; Ito et al., 2001; Hayes et al., 2009; Thornell and Aquilina, 2015; Arrigo, 2017), but opposite effects have been reported (Shemetov et al., 2011). Phosphomimetics of HspB5 have been shown to form smaller but more polydisperse oligomers (Ecroyd et al., 2007; Peschek et al., 2013).

Dynamic sHsp oligomeric structures would be expected to reach equilibrium in a constant environment, but the current literature paints a picture of something more complicated. Since sHsp dynamics are sensitive to many factors, there may be no set equilibrium in the context of an ever-changing cellular environment (Bakthisaran et al., 2015; Haslbeck et al., 2015). This dynamic nature of sHsps can make pinning down a specific structure difficult; and any identified structure of a single oligomeric conformation will likely represent only a snapshot of the evolving assembly. Continuously morphing sHsp oligomers may facilitate the broad recognition of clients that this family of molecular chaperones regulates (Stengel et al., 2010; Fu, 2015).

## sHsp Expression in the Brain

Of the 10 mammalian sHsp proteins, HspB1, HspB5, and HspB8 are expressed in the brain, as well as, HspB6, and HspB7, albeit at lower levels (Quraishe et al., 2008). HspB2 and HspB3 mRNA expression have also been detected at relatively low levels in adult brain (Kirbach and Golenhofen, 2011) (**Table 1**). HspB4 expression appears to be restricted to the eye lens, while HspB9 expression is restricted to testes and heart, and HspB10 expression is restricted to testes, eye, and muscle; these sHsps show no or negligible gene and protein expression in the brain under normal conditions (Quraishe et al., 2008). In addition to the difference in tissue specificity, the regulation of expression is also varied among the HspB family members. Heat shock stress upregulates the expression of HspB1, HspB5, and HspB8 (Chowdary et al., 2004; Kirbach and Golenhofen, 2011). However, *in vitro* HspB8 responds to heat shock in only a subset of cell types (Gober et al., 2003; Chowdary et al., 2004), suggesting that stress-induced expression may not be a ubiquitous phenomenon across all cell types *in vivo*. Brain expression of sHSP family members may be induced by other stressors. For example, HspB1, HspB5, HspB6, and HspB8 were upregulated in response to oxidative stress and hyperosmotic stress in rat hippocampal neurons (Bartelt-Kirbach and Golenhofen, 2014) (**Table 1**). Surprisingly, HspB4, which is not found in normal brain tissues, has been reported to have detectable mRNA and protein expression in cultured primary rat astrocytes, even in the absence of stress induction (Li et al., 2012). It is unclear whether this is reflective of HspB4 expression in a subpopulation of normal brain cells or if expression was induced by culture conditions; either way it is clear that sHsps not normally found in brain tissue have the potential to become expressed in response to stressors, which may include pathological situations (Zhu and Reiser, 2018). HspB1 and HspB5 are more predominantly expressed in glial cells under basal conditions, rather than in neurons (Uhlén et al., 2015; Golenhofen and Bartelt-Kirbach, 2016). Multiple reports demonstrate HspB1 and HspB5 upregulation in neurodegenerative disease (Renkawek et al., 1999; Dabir et al., 2004; Schwarz et al., 2010; Brownell et al., 2012; Liu et al., 2015; Leyns and Holtzman, 2017). For example, in the AD brain, along with other tauopathies including PSP, CBD, and FTDP-17, HspB1 and HspB5 are upregulated in reactive glial cells and can be found to colocalized with glial tau inclusions (Wilhelmus et al., 2006c; Björkdahl et al., 2008; Schwarz et al., 2010). HspB5 upregulation in the astrocytes of ALS patient spinal cord (Iwaki et al., 1992) and HspB1 upregulation in a transgenic mouse model of ALS (Vleminckx et al., 2002) have been reported.

**Figure 2:**
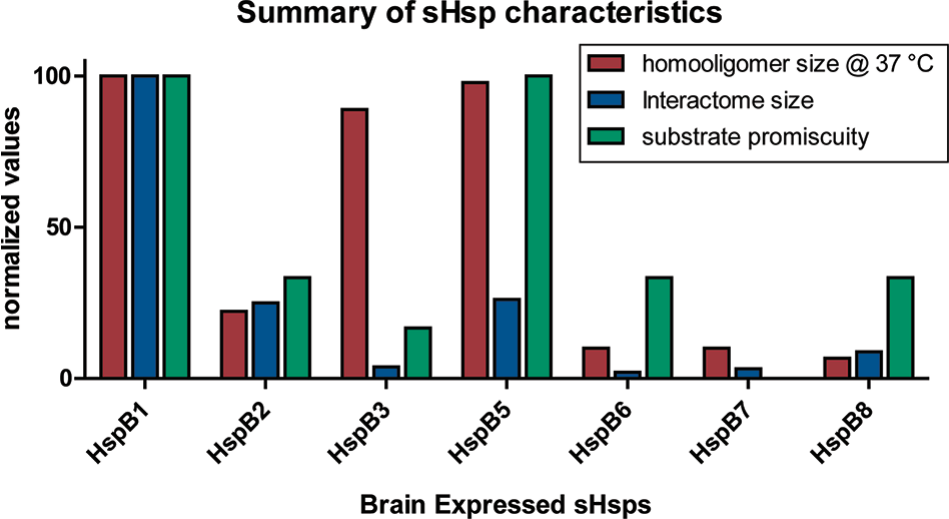
Comparison of specific characteristics of sHsps family members expressed in brain. The average homooligomer size at 37°C was adapted from the relative molecular weights of the peak elution profiles from size exclusion chromatography analysis found in Mymrikov et al., 2017. Chaperone promiscuity was defined as the proportion of six model substrates that demonstrated chaperone activity in a side by side comparison found in Mymrikov et al., 2017. Interactome size was determined by comparison of the number of protein interactors of human sHsps from the BioGRID database version 3.5.170. All values are normalized to HspB1.

**Figure 3:**
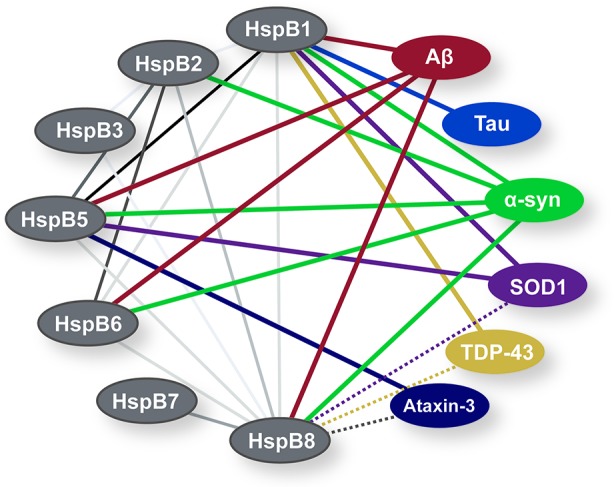
A map of interactions between sHsps expressed in brain and neurodegeneration-associated aggregation-prone proteins discussed in this review. Solid grey lines indicate interactions between sHsps, the darkness of the greyscale lines correlate to the level of evidence for an interaction in BioGRID database 3.5.170. Solid color lines identify physical interactions confirmed *in vitro* between sHsps and aggregation-prone proteins. Dotted color lines indicate presumed interactions based on increased autophagic clearance *via* HspB8/Bag3.

HspB1 and HspB5 share many characteristics that differentiate them from other sHsps (see **Figure 2**). They both readily form higher order homooligomers of 20+ subunits, while other family members generally form smaller oligomers of less than 10 subunits or dimers (Mymrikov et al., 2017). Comparison of the number of human sHsp-interacting proteins also reveals that HspB1 has the largest interactome followed by HspB5 and HspB2 (BioGRID 3.5.170) (**Figure 2**). HspB1 and HspB5 also exhibit chaperone activity across a wider spectrum of model aggregating proteins, compared to other sHsps (Mymrikov et al., 2017) (**Figure 2**). HspB1 and HspB5 subunits comprise ubiquitous large, homooligomeric and, perhaps, heterooligomeric complexes (Kato et al., 1992; Mymrikov et al., 2012; Aquilina et al., 2013; Arrigo, 2013), while other family members appear to function as smaller (under 10 subunits) sHsp oligomers. Alternatively, sHsps that form smaller homooligomers may become incorporated into large sHsp heterooligomers through subunit exchange that correlates with their expression level and sHsp interactome; imbuing new interaction preferences for clients as well as additional proteostasis machinery. Known physical interactions between sHsp family members are summarized in **Figure 3** (BioGRID 3.5.170).

## sHsps Influence Client Proteostatic Fate

sHsps interact with numerous client proteins, especially under stress conditions. Therefore, it can be difficult to determine whether cellular effects of sHsps result from direct interactions with a given client protein or indirectly through modulation of other proteins and/or signaling pathways. Indeed, cytoprotective roles for sHsps have been described, including inhibition of apoptotic signaling pathways, inhibition of neuroinflammation, and buffering against damage from oxidative stress (Mehlen et al., 1996; Préville et al., 1999; Oshita et al., 2010; McGreal et al., 2012; Bakthisaran et al., 2015). Therefore, sHsps will undoubtedly affect cellular proteostasis at multiple levels.

Direct sHsp interactions with misfolded proteins can prevent further irreversible misfolding events and facilitate client triage by other chaperones that more actively influence client fate through specific proteostasis pathways (Hartl et al., 2011; Vos et al., 2011; Ungelenk et al., 2016). Generally, HspB1, HspB4, and HspB5, which are all heat shock responsive, are commonly reported to play a role in substrate refolding (Ehrnsperger et al., 1997; Vos et al., 2010, Vos et al., 2016), while HspB6, HspB7, and HspB8 have been shown to preferentially promote the degradation of clients through autophagy (Fuchs et al., 2009; Vos et al., 2010; Vos et al., 2011; Carra et al., 2013; Vos et al., 2016). However, this simple categorization of HspBs does not hold true in many instances, including many examples discussed in this review. HspB8, the best characterized sHsp in regard to proteostasis mechanisms, forms a complex with the co-chaperone BCL2-Associated Athanogene 3 (Bag3), Hsp70, and C-terminus of Hsc70-interacting protein (CHIP), which appears to drive aggresome formation and promote autophagic degradation of client proteins (Hishiya et al., 2011; Seidel et al., 2012; Minoia et al., 2014; Rauch et al., 2017; Stürner and Behl, 2017). HspB8 overexpression has been reported to lead to enhanced proteasome expression and activity as well as influence its subcellular localization, at least in cardiac tissues (Hedhli et al., 2008). The authors also demonstrated that HspB8 was found in complex with proteasomes together with HspB1 and other chaperones. This may have a general, indirect effect on the degradation of aberrant proteins. HspB1, HspB5, and HspB6 have also been shown to interact with Bag3 (Rauch et al., 2017). In neurodegenerative disease, Bag3 and HspB8 are upregulated in astrocytes (Seidel et al., 2012), which may help clear protein aggregates. HspB6 has been reported to regulate autophagy through direct interaction and stabilization of Beclin 1, a key regulator of autophagosome formation (Liu et al., 2018). Hsp90 inhibition and proteasome inhibition both activate the heat shock response (Bush et al., 1997; Lee et al., 2013, 90), which upregulates HspB1, HspB4, and HspB5 (Bush et al., 1997; Awasthi and Wagner, 2005; Kumano et al., 2012). Proteasome inhibition has also been shown to upregulate HspB8 (Crippa et al., 2010a; Crippa et al., 2010b). Heat shock response has also been shown to promote HspB1 phosphorylation, regulate its oligomerization state (Bolhuis and Richter-Landsberg, 2010), and enhances UPS activity (Gan et al., 2010). Upregulated HspB1 in response to heat shock has been reported to promote proteasomal degradation of certain client proteins (Parcellier et al., 2003; Parcellier et al., 2006). Bag1 competition for Bag3 occupancy in Hsp70/CHIP complexes drives client degradation through the UPS (Lüders et al., 2000; Minoia et al., 2014; Cristofani et al., 2017; Klimek et al., 2017; Rauch et al., 2017; Stürner and Behl, 2017). Aging and oxidative stress correlates with a switch in expression from Bag1 to Bag3 that may shift the client proteins from UPS-mediated degradation to an autophagic pathway (Gamerdinger et al., 2009a; Gamerdinger et al., 2009b; Behl, 2011). Expression and activity of sHsps may influence shuttling misfolded proteins between proteasomal and autophagic degradation pathways, allowing for the alternative system to be utilized when the primary degradation pathway for a given client is impaired or insufficient.

## sHsp Interactions With Amyloidogenic Proteins

Chaperone activity of sHsps was first demonstrated by suppression of stress-induced formation of amorphous aggregates (Horwitz, 1992). The first observation that sHsps also inhibit amyloid fibril formation under physiological conditions *in vitro* was reported using apolipoprotein C-II (Hatters et al., 2001). Since then, a role for sHsp modulation of amyloidogenic protein aggregation has been reported for a variety of client proteins relevant to neurodegeneration (Golenhofen and Bartelt-Kirbach, 2016; Kourtis and Tavernarakis, 2018). Here, we focus on selected amyloidogenic proteins in neurodegeneration with evidence for direct sHsp interactions and/or chaperone activity (**Figure 3**). However, it should be noted that for cellular and *in vivo* applications, direct chaperone activity may be only part of the sHsp-associated mechanisms modifying aggregation or toxicity. Moreover, in disease, while many chaperones still support a healthy environment for the cell (Chen et al., 2018; Grousl et al., 2018), certain chaperone complexes appear to exacerbate the problem of protein misfolding by stabilizing or in some cases promoting the stabilization and accumulation of toxic, prefibrillar oligomers (Tortosa et al., 2009; Blair et al., 2013; Shelton et al., 2017).

### Amyloid β

Amyloid β (Aβ) peptides are cleavage products of the amyloid precursor protein (APP) that are prone to aggregate into toxic oligomers, amorphous aggregates, and fibrils; extracellular deposition of Aβ-rich plaques is considered a hallmark of AD. Multiple sHsps have been reported to affect Aβ, including HspB1, HspB2, HspB5, HspB6, and HspB8. HspB1, HspB2, and HspB6 associate with senile plaques in AD brain tissue (Wilhelmus et al., 2006c). *In vitro* associations between Aβ and HspB1, HspB5, HspB6, and HspB8 have been described (Liang, 2000; Lee et al., 2006; Wilhelmus et al., 2006a; Wilhelmus et al., 2006b). While a connection between HspB1 and Aβ_1-42_ has been reported, little is known about the molecular details of this interaction. What is known is that, in cellular systems, extracellular Aβ_1-42_ induces the expression of HspB1; inversely, exogenous exposure of extracellular HspB1 dose-dependently ameliorated Aβ_1-42_ cellular toxicity (Ojha et al., 2011b). Recombinant protein studies have suggested that the role of HspB1 is to affect Aβ quaternary structure, sequestering toxic oligomers into less toxic, larger aggregates (Ojha et al., 2011b); while other studies have demonstrated a reduction in Aβ aggregation (Wilhelmus et al., 2006a; Yoshiike et al., 2008). A more detailed interaction between HspB5 and Aβ has been described. HspB5 interacts with the hydrophobic core of Aβ_1-40_ and competes for Aβ–Aβ interactions (Narayanan et al., 2006) HspB5 association with Aβ_1-40_ prevented amyloid fibril-associated cellular toxicity (Dehle et al., 2010). HspB5 also binds to Aβ_1-42_ with micromolar affinity and inhibits Aβ fibril elongation and the nucleation of Aβ seeds (Shammas et al., 2011). This work demonstrated that the N-terminal domain of HspB5 was not required for Aβ binding nor inhibition of Aβ fibrillation, whereas the β4–β8 groove of the ACD appears to be crucial for this interaction (Mainz et al., 2015). HspB6 interacts with Aβ and the physical interaction was mapped to residues adjacent to the KLVFF oligomerization domain within Aβ (Cameron et al., 2014). This interaction was strengthened by HspB6 N-terminal domain phosphorylation (Cameron et al., 2014; Cameron et al., 2017. Similar to HspB1, exogenous, extracellular HspB6 also reduced Aβ_1-42_ cellular toxicity. In addition, it was demonstrated that phosphorylation of endogenous HspB6 correlated with an attenuation of Aβ_1-42_ toxicity (Cameron et al., 2017). A direct interaction of HspB8 with Aβ_1-40_ and Aβ_1-42_ was demonstrated using surface plasmon resonance (SPR), with a significantly greater affinity to Aβ_1-40_ with the E22N Dutch mutation that correlates with cerebral amyloid angiopathy (Wilhelmus et al., 2006b). *In vivo* studies using a cross between *HspB2/HspB5* knock-out and mutant APP mice revealed locomotor and sensory deficits, suggesting a negative synergy when these genotypes are combined. However, evaluation of changes to amyloid deposition was not possible, since the knock-out mice did not survive to an age wherein amyloid deposits can be detected (Morrison et al., 2004; Ojha et al., 2011a). More work is needed to fully understand the effects of discrete sHsps or sHsp combinations on Aβ deposition and toxicity.

### Tau

The microtubule-associated protein tau (tau) is an intracellular intrinsically disordered protein that binds to and stabilizes axonal microtubules. Tau is the major constituent of neurofibrillary tangles (NFTs), protein aggregates that form in neurons and contribute to neurodegeneration seen in more than 20 tauopathic diseases, including AD (Götz et al., 2019). Only two sHsps have been characterized for their interactions with tau, HspB1 and HspB5. Interestingly, both HspB1 and HspB5 are upregulated and associated with pathological NFTs in tauopathies (Dabir et al., 2004; Björkdahl et al., 2008). HspB1 has been shown to bind directly to tau and reduce aggregation (Abisambra et al., 2010; Baughman et al., 2018; Freilich et al., 2018; Mok et al., 2018). HspB1 targets early tau aggregates and delays aggregation, but does not inhibit the fibril elongation process once started (Baughman et al., 2018). As recently revealed by nuclear magnetic resonance (NMR), HspB1 recognizes two aggregation-prone regions within the microtubule binding repeat regions of tau *via* both the N-terminal domain and the conserved β4–β8 cleft in the ACD (Baughman et al., 2018; Freilich et al., 2018). Interestingly, this is the same surface used for sHsp dimerization as well as interaction with certain other clients and co-chaperone proteins. The fact that HspB1 binds to several different proteins in the same region, and that this binding is relatively weak, suggests that competitive protein–protein interactions may regulate its chaperone activity toward tau (Freilich et al., 2018). This supports a hypothesis for large, inactive sHsp oligomers that convert to smaller, active oligomers through competitive tau interactions. Phosphorylation of the N-terminal domain, or the introduction of specific pseudophosphorylation mutations, reduces the size of HspB1 oligomeric structures and increases chaperone activity (Hayes et al., 2009; Jovcevski et al., 2015). HspB1 and a pseudophosphorylated mutant both bound tau and reduced or delayed tau aggregation *in vitro*, albeit at different levels (Abisambra et al., 2010; Baughman et al., 2018). Although there are no reports of a direct interaction of tau and HspB8, Bag3 overexpression in neurons reduces tau aggregation; which may implicate a role for HspB8 and autophagy (Lei et al., 2015). Additionally, tau predominantly aggregates in excitatory neurons, with lower Bag3 and HspB8 expression, relative to inhibitory neurons (Fu et al., 2019). HspB1 overexpression was evaluated in brain using a transgenic mouse model of tau aggregation *via* intracranial injection of an adeno-associated viral (AAV) vector (Abisambra et al., 2010). In this model, wild-type HspB1 reduced neuronal tau levels and prevented long-term potentiation (LTP) dysfunction, a cellular correlate of learning and memory. The pseudophosphorylated mutant had opposite effects; increasing neuronal tau levels and failing to prevent the LTP deficit (Abisambra et al., 2010). These results suggest that while reduction of sHsp oligomer size increases chaperone activity *in vitro*, sHsp oligomeric complex dynamics are crucial for proper chaperone activity *in vivo*. Further studies are needed to test other sHsps for their interactions with tau and for their effects in tauopathic mouse models.

### α-Synuclein

α-Synuclein (α-syn) is an intrinsically disordered neuronal protein associated with the regulation of synaptic vesicles (Burré, 2015; Theillet et al., 2016). It is also the primary structural component in Lewy Bodies, large protein aggregates that develop in neurodegenerative synucleinopathies, such as Parkinson’s Disease (PD) or dementia with Lewy Bodies (DLB) (Spillantini et al., 1997; Martí et al., 2003). Point mutations (A30P, E46K, H50Q, and A53T) or whole gene triplication of the *SNCA* gene, encoding α-syn in humans, are associated with the familial forms of early onset PD (Flagmeier et al., 2016; Konno et al., 2016). sHsps interact with multiple species of α-syn, from monomeric partially folded intermediates to fully formed fibrils, modulating both aggregation and cellular toxicity (Cox et al., 2014; Cox et al., 2018). HspB5 was the first sHsp reported to bind α-syn with low affinity and has been known for some time to inhibit amyloid fibril formation (Rekas et al., 2004; Waudby et al., 2010). It has also been reported that α-Syn can interact with at least 5 different sHsps as determined by SPR, with varying affinities; in order of highest affinity for α-syn: HspB5 (K_d_ = ∼60 nM) > HspB1 and HspB6 (∼400 nM) > HspB2 and HspB8 (∼4 µM) (Bruinsma et al., 2011). Each of these five sHsps delayed or inhibited α-syn fibril formation *in vitro*. Remarkably, HspB8 had the most robust inhibition of α-syn fibrillization *in vitro*, but had one of the lowest affinities for α-syn. HspB1 and HspB5 reduced α-syn aggregation, which was attributed to weak transient interactions (Cox et al., 2016), similar to the relationship of HspB1 with tau. This suggests that transient, dynamic sHsp interactions may best ameliorate fibril formation. While HspB5 and HspB8 reduced fibril formation for wild-type α-syn as well as for three familial PD-linked mutants, other sHsps showed more varied effects on discrete α-syn mutants. Surprisingly, HspB2 and HspB6 actually increased the formation of α-syn A53T fibrils (Bruinsma et al., 2011). A recent study found that reduction of the disulfide-bond at the HspB1 β4–β8 dimer interface increased its chaperone activity toward multiple client proteins, including α-syn; perhaps implicating this interface as an important surface for interactions with α-syn (Alderson et al., 2019). This study also demonstrates that the ACD region appears rigid in the dimer, but becomes dynamic and partially unfolded as a monomer (Alderson et al., 2019); this is consistent with the predicted disorder propensity of the HspB1 ACD shown in **Figure 1**. HspB5 expression rescued a rough eye phenotype in a transgenic α-syn fly model (Tue et al., 2012). However, HspB5 overexpression was shown to promote mutant α-syn accumulation in a mouse model of PD through suppression of astrocytic autophagy, while knock-down promoted α-syn clearance (Lu et al., 2019). Additional *in vivo* studies are needed to fully understand the relationship between sHsps and α-syn variants.

### PolyQ Expanded Proteins

Genetic expansion of polyglutamine (polyQ) tracts in certain proteins promotes aggregation into fibrillar structures and the death of neurons in several diseases, including spinocerebellar ataxias (via ataxin proteins), spinal and bulbar muscular atrophy (via the androgen receptor protein), and Huntington’s disease (via the huntingtin protein) (Adegbuyiro et al., 2017). While sHsps have been implicated in an amelioration of aggregate formation in a number of cellular polyQ models, direct interaction between sHsps and polyQ domains has not been reported. This is not surprising as the polar polyQ tract would not directly provide hydrophobic surfaces for sHsp–client interactions. Effects of sHsps on polyQ toxicity may be attributed to direct interactions or secondary effects through an accentuation of autophagy *via* the interaction of HspB8 with the co-chaperone Bag3 (Carra et al., 2008; Carra et al., 2009; Rusmini et al., 2017) or a reduction in oxidative stress (Wyttenbach et al., 2002). HspB5 has been reported to bind to the Josephin domain flanking the polyQ expansion of the ataxin-3 protein, slowing polyQ-driven aggregation (Robertson et al., 2010). A protein engineering strategy similar to the action of HspB5, targeting polyQ-flanking regions with engineered single-chain antibody fragments, also successfully inhibited mutant huntingtin aggregation (Colby et al., 2004). Vos et al. performed an elegant comparison of all 10 sHsps on the aggregation of polyQ expanded huntingtin and ataxin-3, which demonstrated that HspB7 most potently reduced polyQ aggregation in a proteasome-independent manner (Vos et al., 2010). The well-studied and generally promiscuous sHsps, HspB1 and HspB5, were surprisingly not very effective at reducing polyQ aggregation (Vos et al., 2010). It is not yet known whether any of the polyQ huntingtin-active sHsps target a flanking region or act solely through secondary effects. In another study, HspB1 overexpression in a N-terminal huntingtin-fragment transgenic mouse model (R6/2) was unable to alter disease progression or huntingtin aggregation (Zourlidou et al., 2007). However, astrocyte-specific HspB5 overexpression in a full-length huntingtin transgenic model (BACHD) showed neuroprotective effects and slowed disease progression (Oliveira et al., 2016). Moreover, HspB7, ameliorated an eye degeneration phenotype in a fly model expressing polyQ expanded ataxin-3 (Vos et al., 2010) and has yet to be evaluated in mammalian polyQ disease models. More work is needed to understand the direct and indirect actions of sHsps on polyQ expanded aggregates *in vivo*.

### SOD-1 and TDP-43

Superoxide dismutase-1 (SOD-1) is a free radical scavenging metalloenzyme that can contain aggregation-prone mutations linked to familial amyotrophic lateral sclerosis (ALS), a neurodegenerative motoneuron disease. HspB1, HspB5, and HspB8 can all affect the accumulation of this protein. HspB1 and HspB5 can directly interact with SOD-1 *in vitro* and suppress the aggregation of both wild-type and the familial ALS-linked G93A mutant SOD-1 (Yerbury et al., 2013). As with polyQ containing proteins, HspB8 has been shown to facilitate the autophagic removal of misfolded or aggregated SOD-1. In this context, HspB8 interacts with a multimeric complex that also includes Bag3, Hsp70, and CHIP (Crippa et al., 2010b; Crippa et al., 2016a; Crippa et al., 2016b), however there is not yet any direct evidence for direct HspB8 interactions or “holdase” chaperone activity for SOD-1. Similar observations of HspB8-linked enhancement of autophagic clearance have been made for another ALS-linked protein, transactive response DNA-binding protein 43 kDa (TDP-43) (Crippa et al., 2016a), without evidence for a direct interaction. However, there is evidence *via* high throughput co-fractionation data for an interaction of TDP-43 with HspB1 (Wan et al., 2015). Two other studies evaluated overexpression of HspB1 in a transgenic SOD-1^G93A^ model of ALS; one showed no effect on disease progression (Krishnan et al., 2008), while the other showed a modest protection at early stages of disease, which were not sustained and resulted in no improvement at later stages (Sharp et al., 2008). Likewise, *HspB5* knock-down in a transgenic SOD-1 mutant mouse model did not increase SOD-1 aggregation or disease progression (Sharp et al., 2008). However, differential expression of HspB5 ALS transgenic mouse models with varied background correlated with slower disease progression in these animals (Marino et al., 2015). The effects of other sHsp family members on ALS-related aggregates have not been reported.

## sHsp Secretion and Protein Aggregation in the Brain

In addition to intracellular sHsp dynamics, some sHsps have been reported outside of the cell. HspB1, HspB5, and HspB6 have been found in the extracellular environment, in the absence of a traditional secretory signal sequence, and there is evidence for both exosomal and endolysosomal secretion mechanisms (Clayton et al., 2005; Sreekumar et al., 2010; Gangalum et al., 2011; Zhang et al., 2012; Bakthisaran et al., 2015; Batulan et al., 2016). Elevated HspB1 protein levels in serum have been detected in multiple sclerosis (Ce et al., 2011) and in both plasma and cerebrospinal fluid during ischemia (Hecker and McGarvey, 2011). The presence of HspB5 autoantibodies in the serum of AD and PD patients provides additional evidence for the presence of extracellular sHsps in neurodegenerative disease (Papuć et al., 2015; Papuć et al., 2016). sHsp secretion appears to be regulated by post-translational modifications. A phosphorylation deficient HspB1 mutant colocalized with the lysosome-associated membrane protein-1 (LAMP-1) and was preferentially secreted; while a pseudophosphorylated mutant demonstrated decreased secretion and decreased LAMP-1 colocalization (Lee et al., 2012). Evidence for functional roles of these secreted sHsps are emerging (Reddy et al., 2018). In a simple case of direct interaction, sHsps would need to be located outside of cells in order to associate with and reduce toxicity of extracellular misfolded proteins, like Aβ (Ojha et al., 2011b; Cameron et al., 2017); which has been described for HspB1, HspB5, and HspB8 in extracellular, classic, senile plaques in AD brain (Wilhelmus et al., 2006b; Wilhelmus et al., 2006c). However, extracellular sHsps have also been implicated in cell-to-cell communication, signaling, inflammation, and immunity (Binder et al., 2004; Schmitt et al., 2007; Rayner et al., 2008; Reddy et al., 2018), which suggests multiple indirect effects on neuronal health. For example, extracellular HspB5 functions as a stroke neuroprotectant by modulating the immune system (Arac et al., 2011).

Meanwhile, aggregation prone proteins that are traditionally considered to be intracellular, like tau, α-syn, TDP-43, and huntingtin, are also found to be secreted into the extracellular milieu, which may be a mechanism that enables pathological spreading from cell to cell (Hansen et al., 2011; Braak et al., 2013; Babcock and Ganetzky, 2015; Fontaine et al., 2016; Trajkovic et al., 2017; Pérez et al., 2018; Gibbons et al., 2019). There is growing evidence that this cell-to-cell transfer contributes to the anatomical pattern of pathology progression to distinct brain regions that typify specific neurodegenerative diseases (Braak and Braak, 1991; Dickson et al., 2010). Both sHsps and neurodegenerative amyloids are reported to be secreted from cells through similar mechanisms of exosomal release or endolysosomal secretion (Danzer et al., 2012; Batulan et al., 2016; Fontaine et al., 2016; Guix et al., 2018; Katsinelos et al., 2018; Lee et al., 2018; Pérez et al., 2018; Reddy et al., 2018; Sardar Sinha et al., 2018); other mechanisms, including direct transport and tunneling nanotubes, may also contribute (Abounit et al., 2016; Batulan et al., 2016). Physical interactions between sHsps and amyloidogenic proteins may also suggest that this class of molecular chaperones could play a regulatory role in their non-canonical secretion or perhaps buffer against the spread of toxic aggregates to additional cells.

## sHsps and Liquid–Liquid Phase Separation

There is growing evidence that formation of membrane-less organelles can be influenced by some aggregation-prone proteins, including proteins linked to neurodegenerative disease (Patel et al., 2015; Ambadipudi et al., 2017). Membrane-less organelles are viscous, liquid, subcellular compartments containing proteins and often RNA that develop as localized pockets of condensed cellular material formed by a process called liquid–liquid phase-separation (LLPS) (Brangwynne, 2013; Boeynaems et al., 2018; Gomes and Shorter, 2019). Alterations in pH, ion concentrations, temperature, oxidative stress, molecular crowding, and post-translational modifications can trigger molecular supersaturation causing LLPS (Shelkovnikova et al., 2014; Bah and Forman-Kay, 2016; Chen and Liu, 2017; Wang et al., 2017; Boeynaems et al., 2018). Proteins with intrinsically disordered regions can also drive LLPS (Toll-Riera et al., 2012; Brangwynne, 2013; Zhu and Brangwynne, 2015), due to conformational flexibility that allows for a multitude of transient multivalent interactions (Toretsky and Wright, 2014; Boeynaems et al., 2018; Darling et al., 2018). AD, ALS, and frontotemporal dementia-related proteins, such as tau and TDP-43 can form LLPS droplets (Molliex et al., 2015; Patel et al., 2015; Conicella et al., 2016; Ambadipudi et al., 2017; Harrison and Shorter, 2017; Boeynaems et al., 2018; Wegmann et al., 2018; Ambadipudi et al., 2019; Jiang et al., 2019). There is evidence that aberrant membrane-less organelles can become more rigid over time, and this process can be accompanied (or preceded) by the misfolding and pathological aggregation of proteins residing in these organelles (Patel et al., 2015; Ambadipudi et al., 2017; Wegmann et al., 2018).

sHsps share a number of properties with proteins that undergo LLPS, including regions of high intrinsic disorder (**Figure 1**), capability to transiently interact with many substrates, and self-oligomerization (Gomes and Shorter, 2019). The same aggregation-prone motifs in the microtuble-binding regions of tau that are implicated in driving LLPS are also targeted by HspB1 (Baughman et al., 2018; Freilich et al., 2018; Ambadipudi et al., 2019). HspB2 itself has been shown to undergo LLPS, through interactions of the disordered C-terminal domain, which results in the formation of liquid-like compartments in the nucleus that cause impaired nuclear homeostasis and transcription (Morelli et al., 2017). Remarkably, this droplet formation is abrogated by co-expression of HspB3 (Morelli et al., 2017), which forms heterooligomeric complexes with HspB2 (den Engelsman et al., 2009; Clark et al., 2018). Emerging evidence for sHsp residency in membrane-less organelles may suggest additional roles for sHsps in LLPS, including regulation of membrane-less organelle biogenesis and clearance. For example, HspB1, HspB5, and HspB7 have been shown to associate with nuclear speckles (van den IJssel et al., 1998; Bryantsev et al., 2007; Vos et al., 2009). Furthermore, HspB1 and HspB8 are recruited to cytoplasmic stress granules, which are membrane-less organelles (Ganassi et al., 2016). While HspB8 has a role in autophagy, the Hsp70-HspB8-Bag3 complex also plays a role in both the autophagy-independent disassembly of stress granules as well as the extraction of misfolded proteins, known as defective ribosomal products (DRiPs). Additionally, down-regulation of HspB8-Bag3 leads to conversion of stress granules to an aberrant state (Ganassi et al., 2016). HspB1 may also affect stress granule dynamics, but perhaps only after stress granules that have already progressed to an aberrant state (Ganassi et al., 2016).

Membrane-less organelles may provide a microenvironment for the seeding and/or propagation of neurodegeneration-related amyloids. The presence of sHsps in membrane-less organelles may be through a direct interaction with misfolded proteins or due to the intrinsic ability of discrete sHsps to phase separate. Either way, the commingling of neurodegenerative-associated proteins and sHsps in membrane-less organelles may represent a nexus for regulating aggregation. Therefore, modulation of chaperone protein expression or activity may be a potential therapeutic target to treat various neurodegenerative disease pathologies.

## Therapeutic Opportunities

Understanding the interactions and chaperone functions of sHsps with amyloidogenic protein aggregates may better inform small molecule and engineered biologic therapeutic strategies for neurodegenerative proteinopathies.

### sHsp Modulators

Potential therapeutic strategies that aim to modulate endogenous sHsp expression or phosphorylation generally suffer from a lack of specificity for the sHsp family, let alone for discrete sHsps. Heat stress-responsive sHsps can be activated by drugs that generate a challenge to proteostasis, which includes proteasome inhibitors (e.g. Bortezomib), Hsp90 inhibitors (e.g. 17-AAG), and oxidative stress inducers (e.g. terrecyclic acid) (Westerheide and Morimoto, 2005; Powers and Workman, 2007). However, these treatments also induce expression of other molecular chaperone families (e.g. Hsp70 and Hsp40) and are not specific for sHsp activation. Efforts to identify Hsp co-inducers, substances that potentiate stress responses without inducing a primary stress response on their own, may offer improved selectivity (Hargitai et al., 2003; Kasza et al., 2016). Arimoclomol is a co-inducer currently in clinical trials for ALS (NCT00706147, NCT03491462), which may be further enhanced by co-administration with a heat shock response inducer, like Celastrol (Deane and Brown, 2016). As phosphorylation of sHsps can modulate their oligomeric status and chaperone activity, identifying compounds that activate specific kinases or phosphatases that modulate sHsps phosphorylation may also be an attractive therapeutic strategy. In fact, inhibitors of phosphodiesterase 9 were found to induce phosphorylation of HspB6 and reduce Aβ cellular toxicity (Cameron et al., 2017); one of these compounds is currently being tested in an AD clinical trial (NCT00930059).

### sHsp-Targeting Small Molecules

Small molecules that interact with sHsps may be a promising strategy for therapeutics, but the nature of this family of chaperones makes drugability difficult. There are no known small molecule ligands to use as a scaffold to start from. The dynamic and polydisperse nature of these proteins taunt the idea of engineering a high affinity binding drug; indeed, these promiscuous proteins likely have many client binding sites with a variety of conformations. The dimer interface of the ACD, regions within the N-terminal domain, and the IPV motif of the C-terminal domain are important for dimer, higher order oligomer, and client protein interactions. sHsp-client protein interactions compete for oligomeric sHsp–sHsp interactions and activate chaperone activity; these sites have also been proposed as potential drug discovery targets (Arrigo et al., 2007; Jaya et al., 2009; Peschek et al., 2013; Freilich et al., 2018). Oxysterols, like Lanesterol, are an example of potential sHsp-targeting small molecules reported to reverse HspB4 or HspB5 aberrant aggregation, which has been reported as a genetic cause of cataract formation in the eye lens. These compounds appear to reduce the formation of amyloid-like fibers through an interaction with the ACD-dimer interface (Makley et al., 2015; Zhao et al., 2015); however, the high concentrations required for this effect may suggest a lack of specificity for this interaction (Daszynski et al., 2019). sHsp peptide fragments or client binding domains may be promising scaffolds to initiate drug discovery efforts. Small molecules or peptides, with optimally tuned affinities triggering cycling between large oligomers and smaller species, may be more effective “activators” of sHsp chaperone activity than traditional static high affinity binders; as cycling itself has been determined to be crucial for proper chaperone activity *in vivo* with respect to tau aggregation (Abisambra et al., 2010).

### Engineered Molecular Chaperones

Although we generally restricted this review to human sHsps that interact with amyloidogenic proteins involved in neurodegenerative disease, the diversity of sHsps from different organisms, from bacteria to humans, provides a rich set of ACD-containing proteins to explore for aggregation prevention activity (Abisambra et al., 2011). For example, a sHsp from a parasite was shown to be a potent inhibitor of Aβ fibrillation and reduced associated toxicity in a neuroblastoma cell model (Lee et al., 2006). Specific mutant or engineered sHsp variants, with altered oligomeric structure or client interactions, may prove to have increased chaperone activity towards amyloidogenic proteins (Abisambra et al., 2010). Additionally, effective holdase chaperones may be engineered as fusion proteins with domains from other chaperones (foldase or unfoldase) or co-chaperones. Small peptides derived from human HspB4 and HspB5 sequences, termed mini-chaperones, display chaperone-like activity (Raju et al., 2016). One of these constructs reduced cellular toxicity of Aβ (Raju et al., 2018). An important hurdle for eventual clinical translation of engineered molecular chaperones will be delivery to the site of aggregation. Preclinical evaluation of exogenous chaperone expression can be evaluated in mouse models through gene therapy delivery, as used to evaluate tau pathology by our group (Abisambra et al., 2010; Blair et al., 2013; Baker et al., 2017; Shelton et al., 2017); but clinical translation will rely on the continued development of clinical gene therapy or protein transduction domain strategies (Simonato et al., 2013; Naldini, 2015; Guidotti et al., 2017; Raju et al., 2018).

## Author Contributions

JW, AD and VU wrote sections of the manuscript. JW, VU, and LB created the figures, JW and LB edited the manuscript. All authors contributed to the manuscript, read, and approved the submitted version.

## Funding

This work was supported by the National Institute On Aging of the National Institutes of Health under Award Number RF1AG055088. The content is solely the responsibility of the authors and does not necessarily represent the official views of the National Institutes of Health.

## Conflict of Interest Statement

The authors declare that the research was conducted in the absence of any commercial or financial relationships that could be construed as a potential conflict of interest.
